# Warm, Sweetened Milk at the Twilight of Immunity - Alzheimer’s Disease - Inflammaging, Insulin Resistance, *M. paratuberculosis* and Immunosenescence

**DOI:** 10.3389/fimmu.2021.714179

**Published:** 2021-08-05

**Authors:** Coad Thomas Dow

**Affiliations:** McPherson Eye Research Institute, University of Wisconsin-Madison, Madison, WI, United States

**Keywords:** Alzheimer’s disease, autophagy, insulin resistance, *Mycobacterium avium* ss. *paratuberculosis*, MAP, rifampin, Johne’s disease, Crohn’s disease

## Abstract

This article prosecutes a case against the zoonotic pathogen *Mycobacterium avium* ss. *paratuberculosis* (MAP) as a precipitant of Alzheimer’s disease (AD). Like the other major neurodegenerative diseases AD is, at its core, a proteinopathy. Aggregated extracellular amyloid protein plaques and intracellular tau protein tangles are the recognized protein pathologies of AD. Autophagy is the cellular housekeeping process that manages protein quality control and recycling, cellular metabolism, and pathogen elimination. Impaired autophagy and cerebral insulin resistance are invariant features of AD. With a backdrop of age-related low-grade inflammation (inflammaging) and heightened immune risk (immunosenescence), infection with MAP subverts glucose metabolism and further exhausts an already exhausted autophagic capacity. Increasingly, a variety of agents have been found to favorably impact AD; they are agents that promote autophagy and reduce insulin resistance. The potpourri of these therapeutic agents: mTOR inhibitors, SIRT1 activators and vaccines are seemingly random until one recognizes that all these agents also suppress intracellular mycobacterial infection. The zoonotic mycobacterial MAP causes a common fatal enteritis in ruminant animals. Humans are exposed to MAP from contaminated food products and from the environment. The enteritis in animals is called paratuberculosis or Johne’s disease; in humans, it is the putative cause of Crohn’s disease. Beyond Crohn’s, MAP is associated with an increasing number of inflammatory and autoimmune diseases: sarcoidosis, Blau syndrome, autoimmune diabetes, autoimmune thyroiditis, multiple sclerosis, and rheumatoid arthritis. Moreover, MAP has been associated with Parkinson’s disease. India is one county that has extensively studied the human bio-load of MAP; 30% of more than 28,000 tested individuals were found to harbor, or to have harbored, MAP. This article asserts an unfolding realization that MAP infection of humans 1) is widespread in its presence, 2) is wide-ranging in its zoonosis and 3) provides a plausible link connecting MAP to AD.

## Introduction

### AD as a Proteinopathy

Common age-related neurodegenerative diseases are Alzheimer’s disease (AD), Parkinson’s disease (PD), Huntington’s disease (HD) and amyotrophic lateral sclerosis (ALS). While these diseases are distinct clinical entities, at their core, they are all proteinopathies and share a common feature: misfolded and aggregated proteins. For AD, the proteins are amyloid and tau; for PD, synuclein; for HD, Htt; and for ALS, TDP-43 (1). In disease, these proteins lose their physiological functions, aggregate and acquire neurotoxic potential (2). In AD, impairment of protein elimination is central to amyloid accumulation; there is stable production, but inadequate amyloid clearance (3).

### Autophagy

Autophagy is the conserved phylogenetic mechanism critical for intracellular clearance and recycling of aged and/or damaged protein elements occurring in all cell types, including neurons. In the brain, astrocytes and subtypes of microglia play important “janitorial” roles in the phagocytosis and subsequent autophagic elimination of neurotoxic proteins (4).

Autophagy is also critical for the regulation of a wide range of immune responses including innate immunity, inflammation, and antibacterial defense (5, 6). When pathogenic microbes are the target of autophagy the process is called “xenophagy,” a form of selective autophagy (7).

### Infection and Alzheimer’s

Consideration of an infectious contribution to AD is not new. The “usual suspects” are herpes simplex virus 1 (HSV-1), Cytomegalovirus CMV), *Borrelia burgdorferi*, *Chlamydia pneumoniae*, *Helicobacter pylori* and *Porphyromonas gingivalis* (8, 9). Giving credence to a microbial cause of AD is the recognition that amyloid is an antimicrobial peptide (AMP), and the accumulation of this AMP may be reflective of an increasing infectious burden (10, 11). Indeed, the risk of AD appears to increase as the number of concurrent infections increases (12).

**Figure 1:**
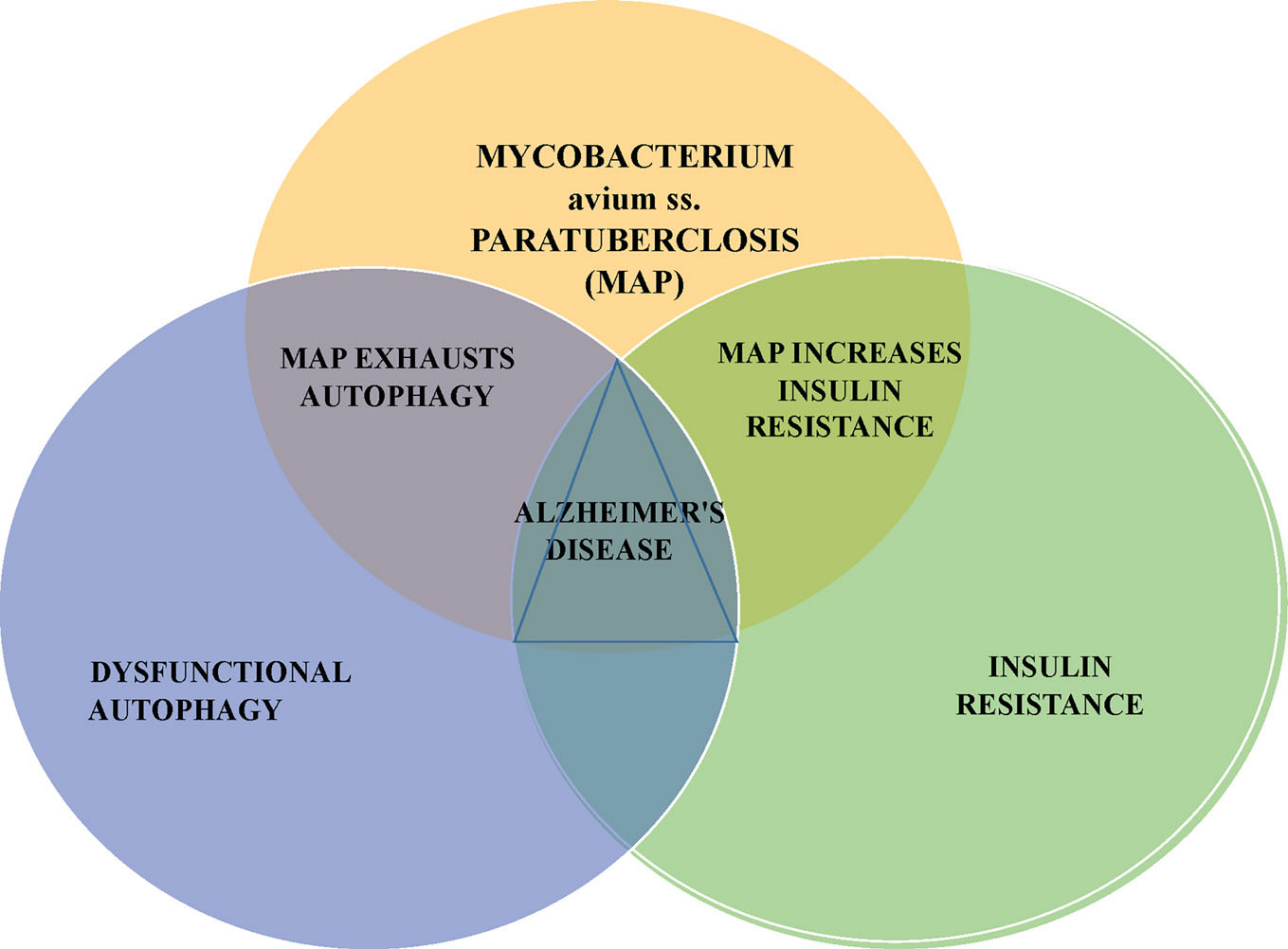
MAP can impair autophagy and promote insulin resistance; these respectively, are the cellular and metabolic changes seen in Alzheimer’s disease. *Mycobacterium avium* subspecies *paratuberculosis* – MAP is a notable bacterium responsible for disease of animals, contamination of food and water and associated with an increasingly long list of human diseases.

This article presents an additional potential AD precipitant, the zoonotic pathogen *Mycobacterium avium* ss. *paratuberculosis* (MAP). MAP has been killing livestock, contaminating food products and has been associated with human inflammatory and autoimmune diseases at a steadily increasing global rate for 100 years (13–15). Moreover, MAP can contribute to cellular and metabolic invariant features of AD; namely dysfunctional autophagy and insulin resistance (**Figure 1**).

Before an in-depth discussion of MAP and AD, it is relevant to review systemic states that are at the intersection of age and AD: inflammaging and immunosenescence.

### Inflammaging

Inflammaging, coined by Franceschi in 2001 (16) refers to a low-grade inflammatory state manifest by 1) expression of genes linked to inflammation 2) higher levels of cytokines in serum and 3) activation of nuclear factor (NF) signaling, the master regulator of inflammatory responses (17–19). Inflammaging is associated with a decline in autophagic capacity impairing cellular housekeeping which leads to protein aggregation and accumulation of dysfunctional mitochondria (20). This age-related phenotype is a risk factor for morbidity and mortality of the elderly and is implicated in the pathogenesis of a number of human maladies including type-2 diabetes, coronary artery disease and Alzheimer’s (21, 22).

### Immunosenescence

Paralleling inflammaging is an age-related reduction in immune competency termed immunosenescence (23). Immunosenescence not only lessens the capacity to respond to infections but also contributes to a number of age-related non-infectious diseases (24, 25). While aging of the immune system has been often described as a shift from naive lymphocytes to memory lymphocytes, that shift is relative; aging itself is not the sole determinant of an accumulation of memory T cells and B cells. For instance, cytomegalovirus infection is associated with an acceleration of immunosenescence (26) as well as increased risk of mycobacterial infection (27, 28).

## Microbes and Alzheimer’s Disease

There is a bi-directional communication, a crosstalk, between the gut microbiota and CNS; the gut-brain-microbiome axis is increasingly understood to play a major role in the pathogenesis of many neurodegenerative disorders including Alzheimer’s disease (AD) (29).

### Amyloid as an Antimicrobial Peptide

There is increasing evidence suggesting that the development of the hallmark pathologic features of AD, amyloid plaque, and tau tangles, can be linked to microbes (30). This is tied to the aforementioned function of amyloid as an antimicrobial peptide (AMP). Studies found that amyloid exerts antimicrobial activity against eight common, clinically relevant microorganisms. Previously unrecognized as an AMP, this contrasting model of amyloid-mediated pathology has important implications for ongoing and future AD treatment strategies (31). Though none of the tested microbes were mycobacteria, it is hoped that this manuscript would prompt future testing of mycobacteria, specifically MAP.

### Microbiome and Alzheimer’s

While there are known factors associated with AD: cerebral ischemia, hypertension, type 2 diabetes metabolic syndrome, high and low body weight, tobacco use and traumatic brain injury (32), change to the microbiome is considered a newly added factor (33–35).

Microbial residents of the intestine are able to modulate the activities of distant sites including the brain *via* bidirectional communication of the GI tract through interactions between the enteric nervous system (ENS) and central nervous system (CNS) (36, 37). Metabolic products of a healthy microbiome are required for the optimal function of the CNS (38).

The largest reservoir of tissue macrophages in the body is contained within the gastrointestinal system. Macrophage response to bacterial presence results in phagocytosis. The response to MAP, however, is blunted due to the fact that MAP prevents phago-lysomal maturation allowing MAP to persist within the macrophage (39).

There is broad acceptance of mycobacterial presence in pulmonary disease, certainly for tuberculosis, and increasingly so for non-tuberculous mycobacteria (40). Conversely, it is unlikely that MAP would be reported in an enteric microbiome report: molecular targets fail to report or under report MAP as it is in low abundance and very difficult to extract from the cell (41, 42); the target genetic sequences are not MAP specific; assays are performed on the gut contents, not the submucosa where MAP thrives (43, 44); MAP overcomes its host, not by sheer numbers but by MAP’s own virulence factors (45).

The proposed mechanism by which MAP promotes AD is the following: MAP persists by inhibition of phagosome maturation, inhibition of toll-like receptor signaling and inhibition of interferon-gamma (IFN-γ) signaling by MAP (13), altered composition of the enteric microbial community by MAP “bio-load” causes and increased mucosal inflammation which loosens epithelial tight junctions, “leaky gut” (46, 47). This permits MAP and/or its byproducts to enter the circulation that contribute to a permeable blood-brain barrier (BBB) allowing their introduction into the brain wherein they produce inflammation and amyloid accumulation/aggregation promoting AD (30).

Although nascent, the study of gut bacteria metabolites upon the brain is an area of active investigation (48). In addition to regulation of both the intestinal barrier and the BBB, several gut microbiota metabolites are able to cross both the intestinal barrier and the BBB presenting a means of communication between the microbiota and the brain. An animal study looking at dietary polyphenols showed minimum absorption with the remainder extensively metabolized by the gut microbiota. Twenty-three polyphenol microbial metabolites were isolated then given intravenously; the brain was found to be a targeted organ for ten of the metabolites (49). Short-chain fatty acids (SCFAs), such as butyrate, are speculated to have a key role in microbiome brain communication; the SCFAs are produced in the gut by bacterial fermentation of dietary fiber (50). These key products of microbiome fermentation may directly or indirectly mediate these interactions *via* signaling routes including immune, endocrine neural and humoral pathways (50).

Humans lack the pathway to produce essential aromatic amino acids; for instance, humans rely on gut bacteria for biosynthesis of phenylalanine, tyrosine, and tryptophan. Multiple studies demonstrate reduced plasma tryptophan levels in probable AD and in clinical AD (51). MAP infection results in upregulation of the enzyme that controls tryptophan metabolism; indoleamine 2,3 –dioxygenase (IDO) levels are increased in MAP infected monocytes, infected ileum and in peripheral blood of infected ruminants. IDO breaks down tryptophan reflected by decreased plasma tryptophan levels and correlated with onset of clinical paratuberculosis (52). This offers a conceptual link between MAP infection and AD.

A recent longitudinal study measuring nine cytokines of 298 cognitively normal elderly found that a higher IFN-γ level was associated with slower cognitive decline independent of amyloid deposition (53). IFNγ plays a central role in immune defense against a variety of intracellular pathogens, including mycobacteria (54). MAP, specifically, inhibits IFN-γ signaling (13), presenting an additional plausible mechanism for the association or MAP and AD.

Only one country, India, has assessed the “bio-load” of MAP in a human population study: over 30% of nearly 30,000 tested positive for MAP: this represents a composite result: 1/3 of serum ELISA tests were positive (past or present exposure), 8.8% of blood samples tested positive by PCR and 22.4 of stool samples tested positive by PCR (55).

## *Mycobacterium Avium* Subspecies *Paratuberculosis* – MAP

### Tuberculosis, Leprosy and Paratuberculosis

There are greater than 140 known *Mycobacterium* species; most of which are considered non-pathogenic or “environmental” (56). *M. tuberculosis* maintains such a prominent place within the species that all others are referred to as non-tuberculous mycobacteria (NTM), many of which have clinical significance particularly in immunocompromised individuals (57).

Based upon genetic sequencing, the mycobacteria responsible for tuberculosis, leprosy and paratuberculosis, are proposed to have gone through an “evolutionary bottleneck” about 10,000 years ago. It is speculated that this was due, in part, to the domestication of and living closely with animals (58). Two of these mycobacteria are well known and studied: tuberculosis has claimed more lives than any other bacterium and a third of the world population is latently infection with *M. tuberculosis* (59). *M. leprae*, responsible for leprosy, is literally biblical in presence and continues today. Official World Health Organization figures report there were more than 202,000 new cases in 2019 (60).

The third mycobacterial agent emerging through this evolutionary bottleneck, MAP, is the long-recognized cause of Johne’s disease. MAP infection known also as paratuberculosis, and recognized worldwide, is an enteric inflammatory infectious disease, mostly studied in ruminant animals: cattle, sheep, and goats.

### Finding MAP

It is very difficult to diagnose the MAP infection in the early, subclinical stage of the disease.

MAP will colonize the intestines of infected animals for years while the animal exhibits no symptoms. However, sub-clinically infected animals continue to shed MAP bacilli in their milk (61) and feces contaminating pastures, the environment, and the food chain (62).

A majority of the dairy herds in the United States and Europe have infected animals within the herd (63). Indeed, according to the USDA, the herd-level prevalence of MAP infection in US dairy herds has markedly increased from 21.6% in 1996 to 91.1% in 2007 (64).

As noted, paratuberculosis is a global disease. Extensive testing in India describes an increasing MAP “bio-load” in cattle (43%), buffalo (36%), goats (23%) and sheep (41%). Moreover, in this same geographic area, 30.8% of 28,291 humans (via serum ELISA, blood PCR and stool PCR) tested positive for MAP (48). Similarly, testing of ruminants in Saudi Arabia found MAP: 26% of sheep, 27% of goats, 30% of cattle and 15% of camels (65).

### MAP in Food

Milk and related dairy products are considered to be the primary source of MAP infection in humans (66); products from pasteurized milk constitute a risk as pasteurization only reduces the MAP load originally present in milk (66, 67). MAP is present in yogurt (68), cheese (69), muscle meat (70) and hamburger (71).

### MAP and Human Disease

Though the link of MAP zoonosis to Crohn’s disease has been controversial for over one hundred years (13), validation of this association has come from studies showing Crohn’s disease resolution with anti-mycobacterial therapy targeted against MAP (72–75). Moreover, MAP is now linked to an increasing list of inflammatory and autoimmune diseases (13, 76). To date, MAP has been causally associated with granulomatous diseases: Crohn’s (77), sarcoidosis (78, 79) and Blau syndrome (80). Through molecular mimicry from mycobacterial heat shock protein (hsp65) (81), MAP induces autoantibodies in autoimmune diabetes (T1D) (82), multiple sclerosis (83, 84), autoimmune thyroiditis (85), lupus (86), rheumatoid arthritis (87, 88) and possibly, Sjogren’s syndrome (89).

## Alzheimer’s: Impaired Autophagy, Insulin Resistance and Mycobacteria

### Alzheimer’s and Impaired Autophagy

Neuronal homeostasis is dependent upon autophagy; the soma of a neuron is the primary site for the degradative pathways while the axon, which extends to synaptic sites as far as a meter away, traffics the lysosome cargo to the soma to complete the degradative process (90). Axonal lysosomes are abundant but are separated from the soma by a selectivity filter that regulates trafficking of the lysosomes to the soma (91). Evidence suggests that amyloid deposits cause a local impairment of retrograde axonal transport of lysosomes leading the further amyloid accumulation.

### Anti-Mycobacterial Agents That Boost Autophagy and Benefit Alzheimer’s

Although an enormous effort has been given to develop AD therapies, there has been little success in finding effective treatments. Currently FDA-approved cholinesterase inhibitors and memantine, while addressing some AD symptoms, lack the ability to slow or stop disease progression (92). Considering the impact of dysfunctional autophagy on both pathogenic mycobacterial infection and AD, perhaps the most compelling argument for mycobacterial involvement in AD can be found in reviewing therapeutic agents that have been found to favorably impact AD *and* mycobacterial infection. A complex interaction between macrophages and pathogenic mycobacterial agents determines the outcome of an infection with these organisms (93). For example, MAP has the ability to retard lysosomal maturation by limiting acidification which improves its virulence and facilitates its survival (45). The remainder of this section identifies therapeutic agents that boost autophagy and by doing so, have potential benefit in both pathogenic mycobacterial infection and AD.

#### mTOR Inhibitors, Rapamycin

The mammalian target of rapamycin (mTOR) signaling pathway is a well described controller of autophagy. Rapamycin and related “rapalogs” are protein kinases that inhibit mTOR (94). As neurons are small, polarized and are post-mitotic they are sensitive to the accumulation of aggregated and damaged cellular proteins and as such are dependent upon efficient autophagy for survival (95). Mounting evidence suggests that AD may be related to mTOR protein synthesis and impaired autophagy (96, 97). Rapamycin is also an effective inhibitor of MAP; its benefit may have unknowingly been an anti-MAP antibiotic (98).

#### Everolimus

Everolimus, as a rapalog, inhibits mTOR and boots autophagy. It has been studied and showed benefit in animal models of AD (99, 100) as well as in mycobacterial infection (101, 102). This includes MAP infection wherein, like with rapamycin, it may act as an anti-MAP antibiotic (98). Both rapamycin and everolimus are from the macrolide antibiotic family of medications, amongst the most potent anti *M. avium* antibiotic families (103).

#### SIRT1 and L-Serine

The silent information regulator 1 (SIRT1) function is linked to cellular metabolism and is activated by L-serine (104). SIRT1 induces autophagy (105) and plays a critical role in controlling mycobacterial disease (106). Activation of SIRT1 reduces the intracellular growth of both drug-susceptible and drug-resistant strains of *M. tuberculosis* and induces phagosome maturation fusion and autophagy in a SIRT1-dependent manner (107). Serine, a dietary amino acid, is currently being studied for early stage AD: ClinicalTrials.gov Identifier: NCT03062449, (108).

#### Calcitriol, Cathelicidin, LL-37

Vitamin D has had an increasingly recognized roll in an expanding variety of diseases including mycobacterial disease and AD; calcitriol causes a dose-dependent inhibition of MAP (109). Calcitriol induces the synthesis of the archetypical antimicrobial peptide LL-37, (cathelicidin), which enhances autophagy (110–113). Specific binding interactions between LL-37 and amyloid complexes may inhibit amyloid aggregation (114).

#### Rifampin and Dapsone

A 1992 epidemiological study revealed that Japanese patients treated with anti-mycobacterial drugs for leprosy had a significantly lower incidence of AD dementia compared with an untreated group (115). Moreover, subsequent histological analyses indicated that non-demented, treated Japanese leprosy patients aged over 70 years showed significantly lower levels of senile plaques in the brain than age-matched non-demented non-leprosy subjects (116, 117). These studies have brought attention to the anti-mycobacterial drugs rifampin and dapsone. Dapsone, an anti-leprosy drug, has neuroprotective effects (118); there is a single reported case of an individual who recovered from AD to mild cognitive impairment, MCI, while on dapsone (119).

Rifampin may have the greatest potential as a repurposed drug for AD (120). Rifampicin is a well-known antibiotic used in the treatment of mycobacterial infections including tuberculosis and leprosy. Rifampin is available for oral and intravenous use; rifampin induces autophagy (121). Clinical trials of therapies that target amyloid-β in patients with AD have revealed that initiating therapy after the onset of clinical symptoms has little effect on cognitive function (122–124) suggesting that preventive therapy should start prior to clinical symptoms. Treatment efforts with rifampin failed in cohorts of mild and moderate AD individuals (125). However, when rifampin was used in preclinical and prodromal AD, it showed preventative effects (126). This amplifies the need for novel plasma biomarkers that identify AD risk which then can be used in clinical trials of individuals with prodromal AD (127). As attractive as repurposed rifampin may be for AD clinical trials, it is notably hepatotoxic (128) and has multiple adverse drug-drug interactions (129).

The nasal route of drug administration has several advantages over oral or intravenous administration, which include non-invasiveness, self-administration, shorter time to onset of effect and higher bioavailability due to avoidance of hepatic first-pass metabolism (130).

Intranasal rifampin delivery has direct access to the brain due to the olfactory and trigeminal neural pathways that connect the nasal mucosa with the brain (131). Moreover, rifampin has advantages for the treatment of AD as it can cross the BBB preventing production of amyloid aggregates as well as amyloid-associated cellular toxicity (132, 133). The permeability-glycoprotein (P-gp) is considered the most important transporter modulating the entry of drugs into the central nervous system (CNS) (134); rifampin, as a potent P-gp inducer, facilitates reduced accumulation of amyloid (135). Thus, intranasal rifampin can access the brain directly *via* ante grade olfactory nerve access, retrograde trigeminal nerve access and *via* a favored permeability status through the BBB.

A further benefit of the anti-mycobacterial agent rifampin, as suggested from animal studies, is that it works favorably in carriers of the ApoE4 allele (136). Also, as with AD, rifampin has a neuroprotective role in Parkinson’s disease (137, 138). MAP has also been associated with Parkinson’s disease (139, 140).

### Alzheimer’s and Insulin Resistance

Accumulating evidence indicates that AD is an age-related, metabolic disease. Impaired cerebral glucose metabolism is an invariant pathophysiological feature in AD and its occurrence precedes cognitive dysfunction and pathological alterations even for decades (141). Compared with age-matched controls, AD individuals show regional glucose metabolism impairment in parieto-temporal lobe, posterior cingulate cortex, and the frontal areas during disease progression (142). Some investigators refer to AD as “Type 3 diabetes mellitus” (143). Insulin has been implicated in clearance of amyloid across the BBB, in tau phosphorylation, and in memory *via* its effects on synaptic function and long-term potentiation (144).

There is a proposed bi-directional relationship between insulin resistance and mycobacterial infection (145, 146); this occurs to the degree that insulin resistance could be considered both a biomarker and risk factor for active mycobacterial infection (147). Insulin resistance is associated with Alzheimer’s disease reflected in a two to five fold increased probability of a type 2 diabetic developing AD (148).

As with rifampin, enhancing brain insulin function with intranasal delivery may be a viable approach to ameliorating AD symptoms and attenuating AD-related pathophysiologic processes (149); this is accomplished without perturbation of the peripheral glucose level as little of the intranasal peptide reaches the peripheral circulation (150). Unlike rifampin, cerebral insulin treatment appears to have less benefit for those carrying the ApoE4 allele(s) (151).

## BCG and Alzheimer’s

### BCG – Background

The primary use of BCG is for the prevention of tuberculosis (152). There is increasing evidence that BCG provides protection against NTM infections (153–155). This extends to leprosy (156). This is unsurprising as BCG, a live attenuated vaccine, shares epitopes with mycobacteria other than tuberculosis (157).

### BCG and Alzheimer’s

A recent population study found an inverse relationship between the incidence of Alzheimer’s disease and BCG vaccination. The populations studied showed a lower prevalence of AD in countries with high BCG coverage. The authors hypothesized that exposure to BCG decreases the prevalence of AD due to a modulation of the immune system. They proposed testing their hypothesis by evaluating bladder cancer patients who received BCG comparing them to bladder cancer patients for whom BCG was not part of their recommended treatment (158). They found that bladder cancer patients treated with BCG were significantly less likely to develop AD compared to those not similarly treated. The mean age at diagnosis of bladder cancer was 68 years. AD was diagnosed at a mean age of 84 years. BCG dramatically reduced the risk of developing AD. Those treated with BCG had four-fold less risk for developing AD compared to patients not treated with BCG. The authors state that confirmation of their retrospective study would support prospective studies of BCG in AD (159). A follow up multi-cohort study again showed protective benefit of intravesicular BCG and risk of AD; it also showed protection against Parkinson’s disease (160). Increasingly appreciated is the protective benefit of not only BCG, but also other live-attenuated vaccines against all-cause infection (161–163).

### BCG for MAP Associated Diseases

BCG vaccination for autoimmune diseases type 1 diabetes (T1D) and multiple sclerosis (MS) have shown benefit in these disparate diseases; both diseases associated with MAP: T1D (164, 165) and MS (166, 167) The positive response to BCG in T1D (168) and MS (169), may be due to a mitigation by BCG of the consequences of MAP infection.

## Discussion

The role of microbial agents in AD is gaining recognition. The current and projected demographics of AD is dire and mandates broad approaches to mitigate the impact AD, not only for individuals and families, but also for global social and health systems. MAP is closely related to the greatest pathogen in human history, *M. tuberculosis*, a microbe that continues to latently infect one third of the world population. MAP may have a role in AD. This article suggests steps to further investigate this potentially fertile line of inquiry: 1) determine population-based MAP “bio-load”, 2) use optimized blood-based biomarkers to determine AD risk, 3) test for MAP in those with elevated AD risk *vs.* healthy controls. Concurrently, interventions could be initiated to 1) eliminate MAP from animals, the environment, and the food chain, 2) initiate clinical trials to test iterations of anti-mycobacterial agents shown to have benefit for AD. Parsimoniously, when searching for new directions in the efforts against AD, look at the MAP.

## Author Contributions

The author confirms being the sole contributor of this work and has approved it for publication.

## Conflict of Interest

The author declares that the research was conducted in the absence of any commercial or financial relationships that could be construed as a potential conflict of interest.

## Publisher’s Note

All claims expressed in this article are solely those of the authors and do not necessarily represent those of their affiliated organizations, or those of the publisher, the editors and the reviewers. Any product that may be evaluated in this article, or claim that may be made by its manufacturer, is not guaranteed or endorsed by the publisher.
